# ASO Author Reflections: Nevus-Associated Melanoma: A Prognostically Distinct Entity

**DOI:** 10.1245/s10434-025-16999-2

**Published:** 2025-02-18

**Authors:** Nazia Riaz, Roger Olofsson Bagge

**Affiliations:** 1 Department of Surgery, Institute of Clinical Sciences, Sahlgrenska Academy, University of Gothenburg, Gothenburg, Sweden; 2 Department of Surgery, Sahlgrenska University Hospital, Gothenburg, Sweden

## Past

Cutaneous melanomas arising in association with preexisting nevi, called nevus associated melanomas, are encountered in up to 30% of the histological specimens, although these figures may vary considerably. The biological and clinical consequences of this observation remain incompletely understood. Like melanomas, BRAF^V600E/K^-activating mutations are frequently found in melanocytic nevi. Additionally, studies investigating genetic predisposition and somatic mutations suggest that melanocytic nevus could serve as a potential precursor lesion in melanoma histogenesis.^1^ Yet, a vast majority of melanocytic nevi are growth arrested, suggesting an interplay of alternate homeostatic mechanisms such that tumor suppression prevails.^2^ Nonetheless, accumulating evidence supports the notion that nevus-associated melanoma displays clinicopathological features uniquely distinct from de novo melanomas. Prior clinical studies have shown inconsistent results regarding the prognostic implications of nevus associated melanomas compared with de novo melanomas.^3^^,^^4^ Hence, we sought to investigate the prognostic implications of preexisting nevi in cutaneous melanoma utilizing a large multi-institutional cohort from the Sentinel Lymph Node Working Group database.

## Present

Using a prespecified analytical approach, our results demonstrate that compared to de novo melanomas, nevus-associated melanomas show a significant association with relatively younger age at diagnosis, superficial spreading subtype, and less aggressive pathological features. Importantly, patients with nevus-associated melanomas experienced a significantly better prognosis compared with those with de novo melanomas as evidenced by improved relapse-free, melanoma-specific, and overall survival, even after adjusting for known prognostic factors. However, nevus associated melanomas did not show any predictive association with the status of sentinel lymph nodes.^5^

## Future

As highlighted in our study, nevus associated melanoma has an independent and favorable impact on prognosis. Prospective validation of these findings in related cohorts are necessary to confirm these results. Omics-based investigations may further enhance our understanding of the biological role of preexisting nevi in cutaneous melanoma.
